# Understanding how organisational culture supports patient involvement in a national research agency

**DOI:** 10.1093/eurpub/ckac131.538

**Published:** 2022-10-25

**Authors:** I Cleemput, M Dauvrin, L Kohn, P Mistiaen, W Christiaens, C Léonard

**Affiliations:** Belgian Health Care Knowledge Centre, Brussels, Belgium

## Abstract

**Background:**

The Belgian Health Care Knowledge Centre (KCE) formally involves stakeholders in its researches since 2012. Patients are treated as one stakeholder amongst others, but it is recognized that patient involvement (PI) requires a different approach. The success of implementing PI depends, however, on the organizational culture towards PI.

**Objectives:**

The objective of this study was to map the PI culture at KCE in the context of the development of organization-wide supported position statements about PI.

**Methods:**

A nominal group technique was used to measure the PI culture at KCE. Arguments for and against PI and conditions for PI in different phases of the research process were collected. A literature review and interviews fed the draft position statements, for which support was assessed by means of a two-round Delphi process.

**Results:**

Arguments in favour of PI in research related to the relevance of the scope, expertise with data collection, bringing in fresh ideas for study design, access to survey participants, validation of data analyses, adherence to recommendations. Disadvantages and risks included the lack of scientific knowledge of involved patients, resources requirements, conflicts of interest, and heterogeneity within patient populations. Conditions for meaningful PI referred to measures mitigating the identified disadvantages. Eighteen position statements supported by KCE could be formulated.

**Conclusions:**

The KCE culture seems predominantly positive towards PI, although attitudes vary between researchers. KCE recognizes the potential value of PI in research, but considers the level of involvement to be contingent on the topic and phase in the research process.

**Key messages:**

• Organizational culture towards patient involvement is a driver of successful researches.

• 18 position statements supported implementation of patient involvement in a national health agency.

